# Proteome heterogeneity and malignancy detection in pancreatic cyst fluids

**DOI:** 10.1002/ctm2.506

**Published:** 2021-08-23

**Authors:** Sheng Pan, Randall E. Brand, Lisa A. Lai, David W. Dawson, Timothy R. Donahue, Stephen Kim, Natalia I. Khalaf, Mohamed O. Othman, William E. Fisher, Mary P. Bronner, Diane M. Simeone, Teresa A. Brentnall, Ru Chen

**Affiliations:** ^1^ The Brown Foundation Institute of Molecular Medicine University of Texas Health Science Center at Houston Houston Texas USA; ^2^ Department of Integrative Biology and Pharmacology McGovern Medical School University of Texas Health Science Center at Houston Houston Texas USA; ^3^ Department of Medicine University of Pittsburgh Pittsburgh Pennsylvania USA; ^4^ Division of Gastroenterology Department of Medicine the University of Washington Seattle Washington USA; ^5^ Department of Pathology and Laboratory Medicine David Geffen School of Medicine, UCLA Los Angeles California USA; ^6^ Jonsson Comprehensive Cancer Center David Geffen School of Medicine, UCLA Los Angeles California USA; ^7^ Department of Surgery David Geffen School of Medicine, UCLA Los Angeles California USA; ^8^ Division of Digestive Diseases David Geffen School of Medicine, UCLA Los Angeles California USA; ^9^ Section of Gastroenterology and Hepatology Department of Medicine Baylor College of Medicine Houston Texas USA; ^10^ Department of Surgery Baylor College of Medicine Houston Texas USA; ^11^ Department of Pathology University of Utah Salt Lake City Utah USA; ^12^ Department of Surgery New York University New York New York USA; ^13^ Perlmutter Cancer Center New York University New York New York USA

Dear Editor:

Pancreatic cyst neoplasms (PCNs), such as intraductal papillary mucinous neoplasms (IPMNs) and mucinous cystic neoplasms (MCNs), represent one of the main dysplastic precursor lesions that could give rise to invasive pancreatic carcinoma.^1^, ^2^ While guidelines have been suggested to assist in the diagnosis and management of PCNs, including resection and surveillance recommendations,^3^, ^4^ clinical management of cyst lesions remains imprecise due to difficulties in accurately detecting high‐risk or invasive lesions and uncertainty in predicting the malignant potential of these lesions. Current diagnostic evaluations of PCNs, including cyst size and morphology, worrisome features, main pancreatic duct dilation, CA19‐9, cytology, and cyst fluid analysis (CEA, amylase), can discriminate between mucinous and non‐mucinous cysts and classify cyst types with some certainty, but they do not provide a definite clinical diagnosis of PCNs with high‐risk or invasive lesions.^5^, ^6^ A biomarker test that can effectively assist PCN risk stratification and treatment decision‐making would be clinically valuable. In this study, we applied a spectral library‐based proteomic platform^7^ to interrogate cyst fluid proteomes of various PCNs in the context of biomarker development.

Cysts fluids from patients with various PCNs can be highly heterogeneous. The physical appearance of the cyst fluids acquired from IPMN, MCN, serous cystic adenomas, and pseudocyst appeared to be quite different (Figure 1A). Their dissimilarities were reflected in the diverse protein concentrations and the number of proteins identified therein (Figure 1B). Figures 1C and D exemplify the proteomic overlap among the cyst fluids shown in Figure 1A. Although specific proteins can be accurately interrogated with mass spectrometry (Figure 1E), the immense heterogeneity in cyst fluid proteomes among different individuals or cyst types has posed analytical challenges in developing robust biomarkers to assist in PCN diagnosis.

**FIGURE 1:**
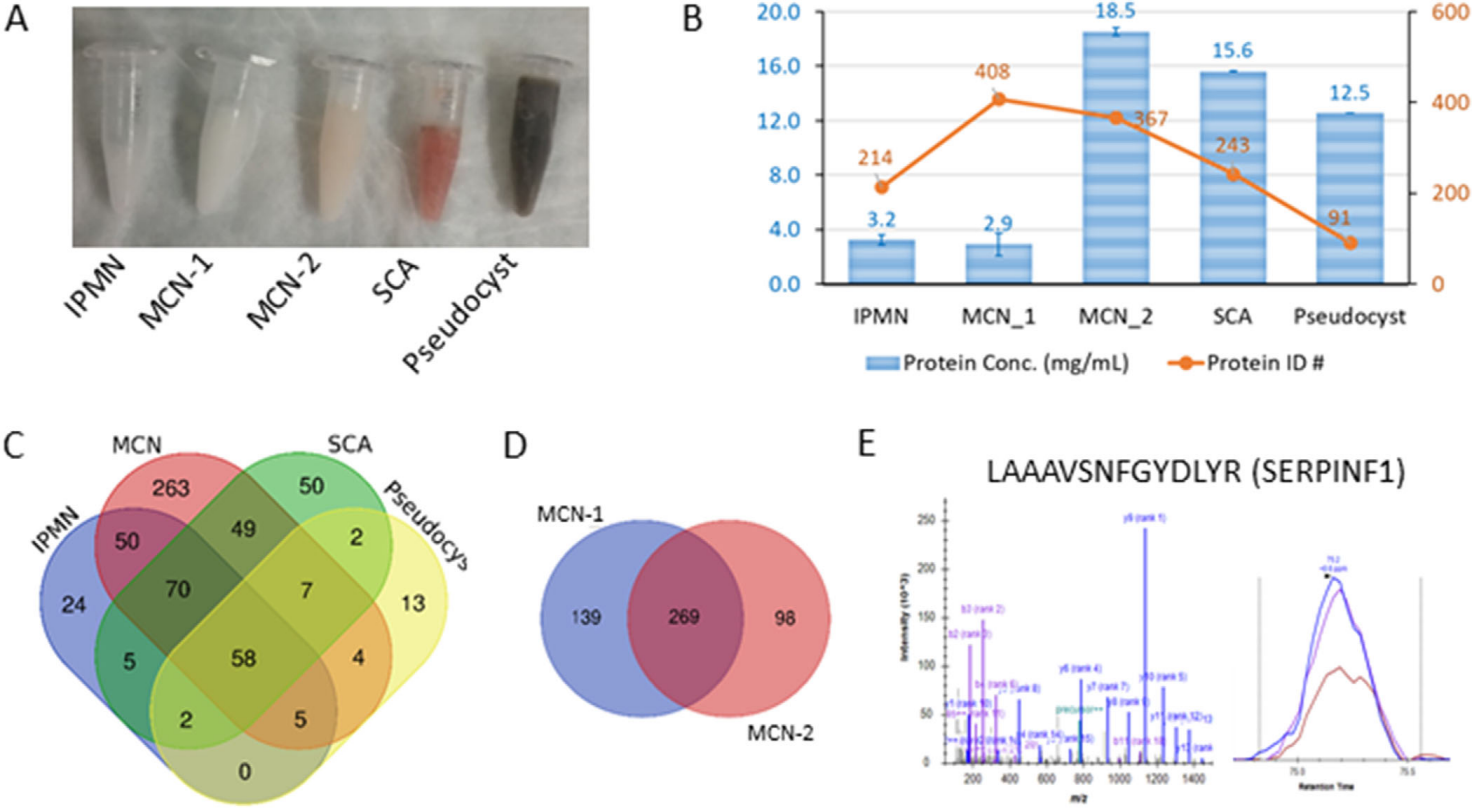
Proteome heterogeneity of pancreatic cyst fluids. (A) Physical appearances of different cyst fluids: intraductal papillary mucinous neoplasms (IPMN), mucinous cystic neoplasms (MCN), serous cystic adenomas (SCA), and pseudocyst. (B) Protein concentrations (blue bars) and the number of proteins identified (orange nodes) in the cyst fluid specimens. No correlation was observed between protein concentrations and the number of proteins identified. (C) Overlap of the proteins identified in the cyst fluid samples. (D) Overlap of the proteins identified in the two MCN samples. (E) Exemplification of peptide identification and quantification using peptide LAAAVSNFGYDLYR from protein SERPINF1

A cohort of 20 cyst fluid specimens was analyzed to interrogate the proteome of cyst fluids and identify cancer‐associated proteome alterations for developing effective strategies for malignancy detection. These pathoclinically well‐defined patient samples comprised 12 IPMNs and eight MCNs, including nine cases with histologically confirmed carcinoma or high‐grade dysplasia (HGD) and 11 cases with benign or low‐grade dysplasia (LGD) (Table S1). While the majority of Carcinoma/HGD cases had a higher protein concentration compared to Benign/LGD, the difference was not statistically significant (Figure 2A). Using the spectral library‐based platform, which integrated proteomic discovery with targeted analysis, we identified >2400 proteins in these cyst fluid specimens. Functional analysis indicated that many cyst fluid proteins were involved in cell‐cell adhesion, proteolysis and innate immune response, more than 30% of the proteins were related to signaling, and ∼67% and ∼53% were subject to changes due to polymorphism or alternative splicing, respectively (Figure S1).

**FIGURE 2:**
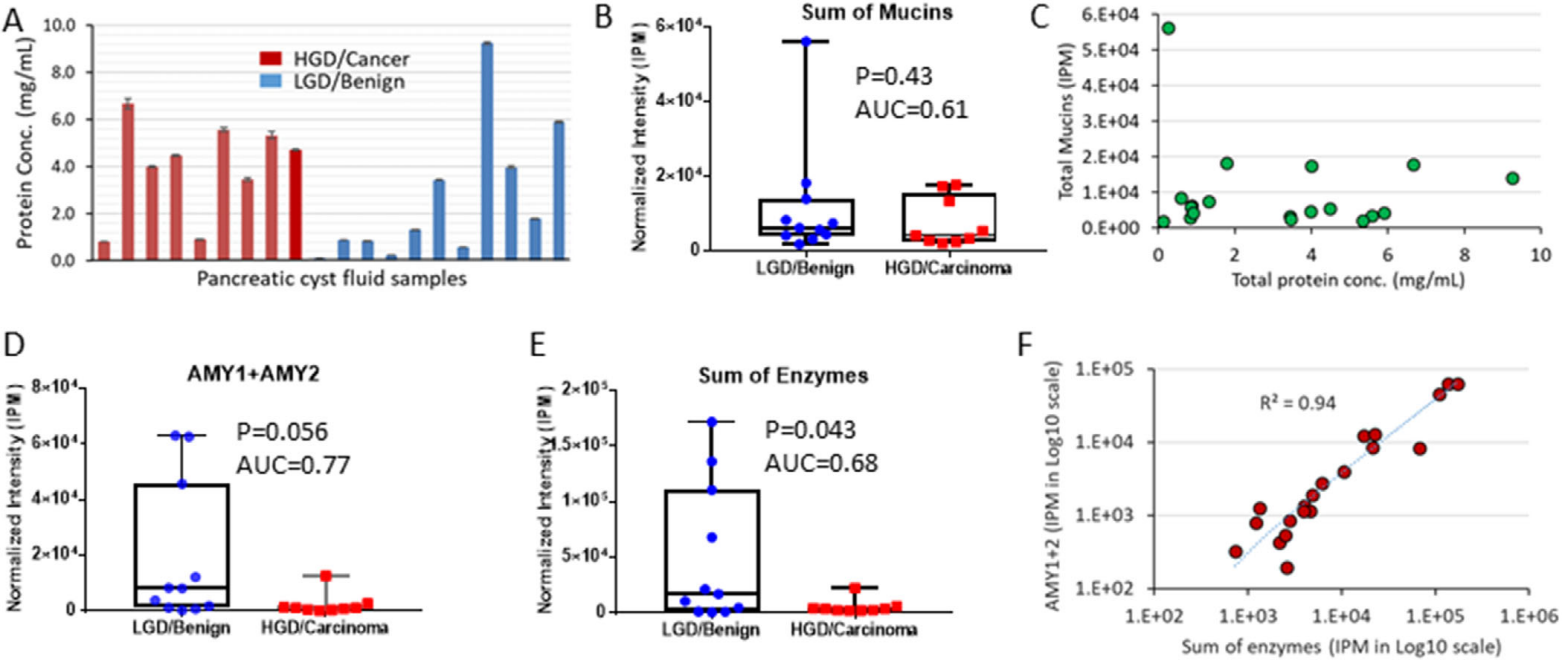
Exemplification of some characteristics of cyst fluid proteomes from the Carcinoma/HGD patients in comparison to the Benign/LGD controls. (A) The protein concentrations of the cyst fluid samples. (B) Comparison of the sum of mucins identified in the Benign/LGD and Carcinoma/HGD groups. (C) Correlation of total mucins with overall protein concentration. (D) Comparison of amylases (AMY1 + AMY2) identified in the Benign/LGD and Carcinoma/HGD groups. (E) Comparison of the sum of enzymes (amylases, chymotrypsin‐like elastases, carboxypeptidases, chymotrypsinogens, pancreatic triacylglycerol lipases, and trypsins) identified in the Benign/LGD and Carcinoma/HGD groups. F) Correlation of amylases (AMY1+AMY2) abundance with the sum of enzymes

Mucins are an important group of proteins relevant to pancreatic cancer, and part of the antigen complex in CA19‐9 detection. A large number of mucins were identified in the cyst fluid samples, including MUC1, MUC2, MUC3A, MUC3B, MUC4, MUC5AC, MUC5B, MUC6, MUC13, MUC16, MUC17, and MUC19 (Figure S2). However, in contrast to what was previously suggested,^8^ our data did not support mucins as an effective biomarker for malignancy detection based on the study cohort. In general, there were not significant mucin differences between the Carcinoma/HGD and Benign/LGD groups. This observation may partly explain why CA19‐9 does not work well as a cyst fluid biomarker for pancreatic cancer detection. At an overall level, the total mucin content was not significantly different between the two groups, nor did it correlate with the total protein concentrations (Figures 2B and C).

On the other hand, a large number of pancreas secreted enzymes were found decreased in the Carcinoma/HGD group, including amylases (AMY), chymotrypsin, n‐like elastases, carboxypeptidases, chymotrypsinogens, pancreatic triacylglycerol lipases, and trypsins (Figure S3). AMY have been a clinical biomarker to distinguish pseudocysts. Although the changes in AMY and other enzymes between the two groups were noteworthy (Figures 2D and E), these enzymes were also highly heterogeneous among the individuals within the same group, diminishing their value for malignancy detection. Furthermore, a strong correlation between AMY and the sum of other enzymes was observed (Figure 2F), suggesting that the decrease of the enzymes in the Carcinoma/HGD group might likely be due to the diminution or damage of acinar cells by a tumor.

Using a selected group of pancreatic cancer‐associated proteins with an elevated concentration in Carcinoma/HGD (*p* < 0.05) and pancreatic enzymes, a principal component analysis was able to clearly seperate the Carcinoma/HGD cases from Benign/LGD cases (Figure 3A). To minimize the influence of inter‐sample heterogeneity for malignancy detection, an internal ratio‐based biomarker approach was employed using the abundance of AMY for normalization (protein / AMY × 100). Six best‐performed proteins, including CEACAM5, FN1, GSN, HSPA5, ITIH4, and SERPINF1, were selected for further evaluation. The receiver operating characteristic (ROC) analyses indicated that these AMY‐normalized proteins all had an area‐under‐the‐curve (AUC) value ≥0.92. The patients’ age, gender, mucinous cyst types, or diabetic status did not appear to cause confounding effects on the quantification of these proteins (Figure S4). By excluding candidates that were highly correlated with an *R* > 0.70 (Figure 3B), a composite proteomic signature consisting of CEACAM5/AMY, SERPINF1/AMY, and HSPA5/AMY was proposed. Using logistic regression analysis, the composite signature had an AUC value of 0.99 (Figure 3C) and was able to predict eight out of nine cases of Carcinoma/HGD and all 11 cases of Benign/LGD (Figure 3D), affording sufficient precision to rule in HGD or malignant PCNs for further examination.

**FIGURE 3:**
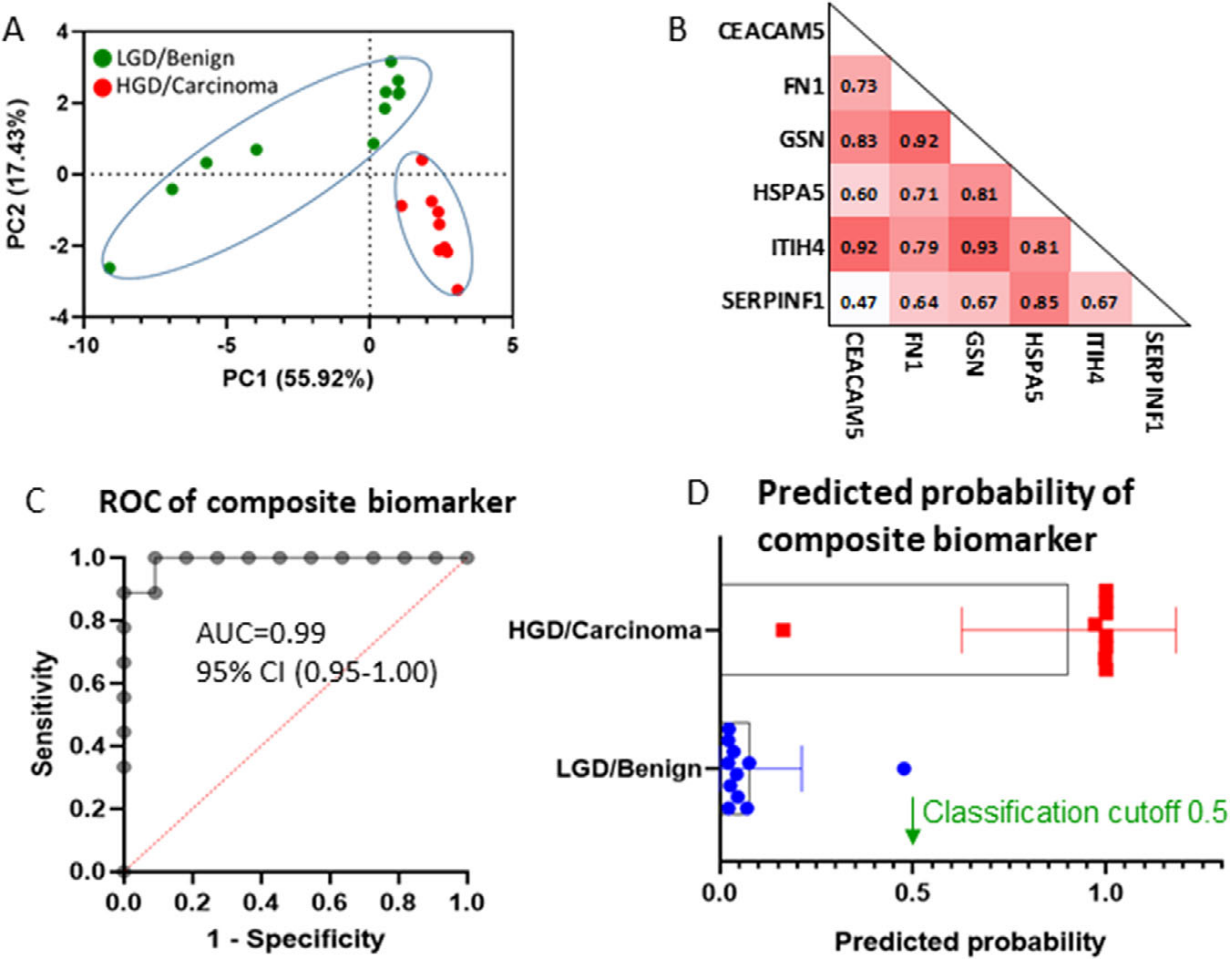
Cyst fluid protein candidates to distinguish Carcinoma/HGD group from Benign/LGD group. (A) Principal component analysis (PCA) to distinguish Carcinoma/HGD from Benign/LGD. The proteins included are CEACAM5, FCGBP, FN1, GSN, HP, HSPA5, ITIH4, KNG1, MYH9, SERPINF1, and pancreatic enzymes, including AMY1A, AMY2A, AMY2B, CELA2A, CELA2B, CPA1, CPB1, CTRB1, CTRB2, PNLIP, PRSS1, and PRSS2. (B) Correlation analysis of amylase‐normalized protein CEACAM5, FN1, GSN, HSPA5, ITIH4, and SERPINF1. (Spearman's correlation coefficient *R* interpretation: 0.3–0.5 fair, 0.5–0.7 moderate, 0.7–0.9 very strong, 1 perfect). C) Receiver operating characteristic (ROC) curves of the ratio‐based composite biomarker (CEACAM5/AMY, SERPINF1/AMY, and HSPA5/AMY). (D) Predicted probability of composite biomarker using logistic regression. The standard cutoff of 0.5 was used to determine a positive result

CEACAM5^9^ and SERPINF1^7^ have been previously associated with pancreatic cancer detection, and CEACAM5 is the major antigen for the current clinical CEA test. HSPA5 is a master regulator of the unfolded protein response under endoplasmic reticulum stress and was implicated in acinar‐to‐ductal metaplasia and PanIN development in the KPC mouse model.^10^ Detection of significantly higher HSPA5 in the cystic fluid could be a manifestation of PCN malignancy transformation if further validated. The TCGA tissue RNA expressions of CEACAM5, SERPINF1, and HSPA5 in pancreatic cancer^11^ are illustrated in Figure S5.

Compared to the current clinical CEA assay (87.5% accuracy),^12^ the AMY‐normalized composite signature demonstrated a significantly improved accuracy in detecting malignant IPMNs/MCNs, and therefore merited further studies for its clinical value for risk stratification in cyst lesion management.

## CONFLICT OF INTEREST

The authors declare no conflict of interest.

## ETHICS APPROVAL

The study protocol was approved by the Institutional Review Boards at the University of Pittsburgh, the University of California at Los Angeles. Informed consent was obtained from each patient for pancreatic cyst fluid collection.

## AUTHOR CONTRIBUTIONS

Sheng Pan, Ru Chen, Teresa A. Brentnall, and Randall E. Brand planned the study. Sheng Pan, Lisa A. Lai, David W. Dawson, Mary P. Bronner, and Ru Chen conducted the study. Sheng Pan, Ru Chen, Teresa A. Brentnall, Randall E. Brand, David W. Dawson, Timothy R. Donahue, Stephen Kim, Mary P. Bronner, and Diane M. Simeone provided the resources. All authors participated in preparing and reviewing the manuscript.

## DATA AVAILABILITY STATEMENT

The data that support the findings of this study are available from the corresponding author upon reasonable request.

## Supporting information

**Table 1**. Demographic and clinical data of IPMN/MCN study cohort
**Figure 1**. Functional annotation using DAVID (The Database for Annotation, Visualization, and Integrated Discovery).
**Figure 2**. The levels of mucins in the pancreatic cyst fluid specimens.
**Figure 3**. The levels of individual enzymes in the pancreatic cyst fluid specimens.
**Figure 4**. The quantification of amylases‐normalized CEACAM5, FN1, GSN, HSPA5, ITIH4, and SERPINF1 versus patients’ age (A), gender (B), mucinous cyst types (C), or diabetic status (D). No significant correlations were observed between ratio‐based quantifications and the patients' age, gender mucinous cyst types, or diabetic status
**Figure 5**. Tissue RNA expression analysis in pancreatic cancer using TCGA database for CEACAM5, SERPINF1, and HSPA5. Using the TCGA RNA‐seq dataset available from v19.1 ProteinAtlas.org, the RNA expression of CEACAM5, SERPINF1, and HSPA5 were evaluated, and only CEACAM5 was significantly linked to tumor stages and/or patient survival time. NS: statistically non‐significant.

Supporting information
