# Supplementary material for: Proteome heterogeneity and malignancy detection in pancreatic cyst fluids
Source: Clin Transl Med. 2021 Aug 23;11(8):e506. doi: 10.1002/ctm2.506 (PMC8382978; doi:10.1002/ctm2.506)
Supplement: Supplementary file 1 — Table 1. Demographic and clinical data of IPMN/MCN study cohort Figure 1. Functional annotation using DAVID (The Database for Annotation, Visualization, and Integrated Discovery). Figure 2. The levels of mucins in the pancreatic cyst fluid specimens. Figure 3. The levels of individual enzymes in the pancreatic cyst fluid specimens. Figure 4. The quantification of amylases‐normalized CEACAM5, FN1, GSN, HSPA5, ITIH4, and SERPINF1 versus patients’ age (A), gender (B), mucinous cyst types (C), or diabetic status (D). No significant correlations were observed between ratio‐based quantifications and the patients' age, gender mucinous cyst types, or diabetic status Figure 5. Tissue RNA expression analysis in pancreatic cancer using TCGA database for CEACAM5, SERPINF1, and HSPA5. Using the TCGA RNA‐seq dataset available from v19.1 ProteinAtlas.org, the RNA expression of CEACAM5, SERPINF1, and HSPA5 were evaluated, and only CEACAM5 was significantly linked to tumor stages and/or patient survival time. NS: statistically non‐significant. [file CTM2-11-e506-s002.pdf]

Supplemental Table 1. Demographic and clinical data of IPMN/MCN study cohort

Specimen Type	Diagnosis	Cancer stage - if applicable	Gender	Race	Age	Smoker status	Diabetic
Surgical Cyst Fluid	IPMN with LGD		Female	White	65	Past	No
EUS Cyst Fluid	IPMN with LGD		Male	White	77	Never	Yes
EUS Cyst Fluid	IPMN with LGD		Female	Black/African America	82	Past	Yes
Surgical Cyst Fluid	IPMN degenerated into adenocarcinoma	T1N0M0; Stage IA surg	Female	White	84	Never	Yes
Surgical Cyst Fluid	MCN with HGD		Female	White	58	unknown	unknown
EUS Cyst Fluid	MCN with LGD		Male	White	56	Never	No
Surgical Cyst Fluid	IPMN degenerated into adenocarcinoma	T1N0M0; Stage IA surg	Male	White	65	Past	No
EUS Cyst Fluid	IPMN with LGD		Male	White	61	Never	No
Surgical Cyst Fluid	IPMN with HGD		Male	White	67	Past	No
Surgical Cyst Fluid	IPMN with HGD		Male	White	74	Never	No
Surgical Cyst Fluid	IPMN with LGD		Female	White	75	Never	Yes
Surgical Cyst Fluid	IPMN degenerated into adenocarcinoma	T1N0M0; Stage IA surg	Female	White	69	Past	Yes
Surgical Cyst Fluid	MCN with LGD		Female	White	56	Never	Yes
EUS Cyst Fluid	MCN with LGD		Female	White	47	Current	No
Surgical Cyst Fluid	MCNwith LGD		Female	White	42	Current	Yes
Surgical Cyst Fluid	MCN degenerated into adenocarcinoma	T1N0M0; Stage IA surg	Female	White	34	Never	No
EUS Cyst Fluid	MCN with LGD		Female	Black/African America	48	Never	No
Surgical Cyst Fluid	IPMN degenerated into adenocarcinoma	T3N1M0; Stage IIB surg	Male	White	78	Past	Yes
Surgical Cyst Fluid	IPMN with LGD		Female	White	51	Past	No
Surgical Cyst Fluid	MCN with HGD		Female	White	42	Never	No

Term	Count	%	PValue	FDR
GO:0006508~proteolysis	155	6.31879331	1.11E-24	2.12E-21
GO:0007155~cell adhesion	119	4.85120261	1.01E-12	1.94E-09
GO:0045087~innate immune response	101	4.11740726	1.80E-08	3.46E-05

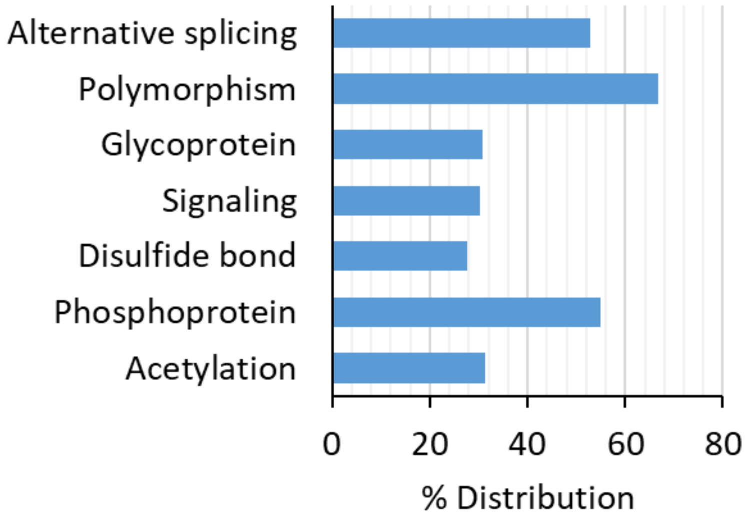

Supplemental Figure 1. Functional annotation using DAVID (The Database for Annotation, Visualization and Integrated Discovery).

Ref: Huang DW, Sherman BT, Lempicki RA. Systematic and integrative analysis of large gene lists using DAVID Bioinformatics Resources. Nature Protoc. 2009;4(1):44-57.

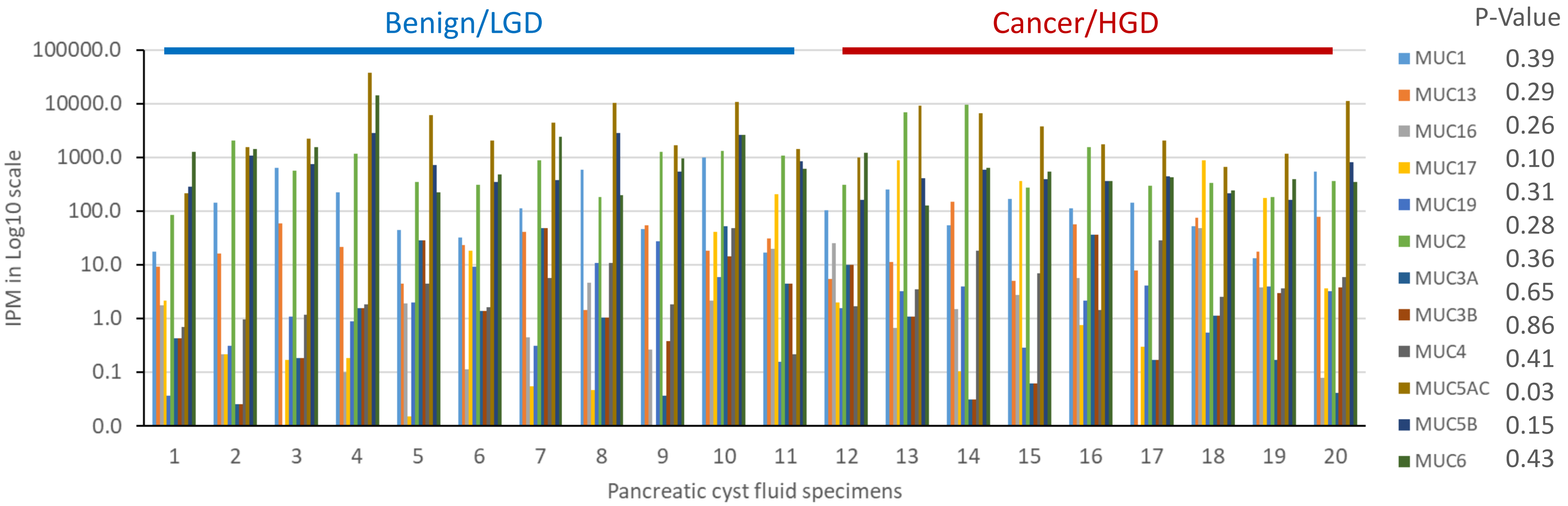

Supplemental Figure 2. The levels of mucins in the pancreatic cyst fluid specimens.

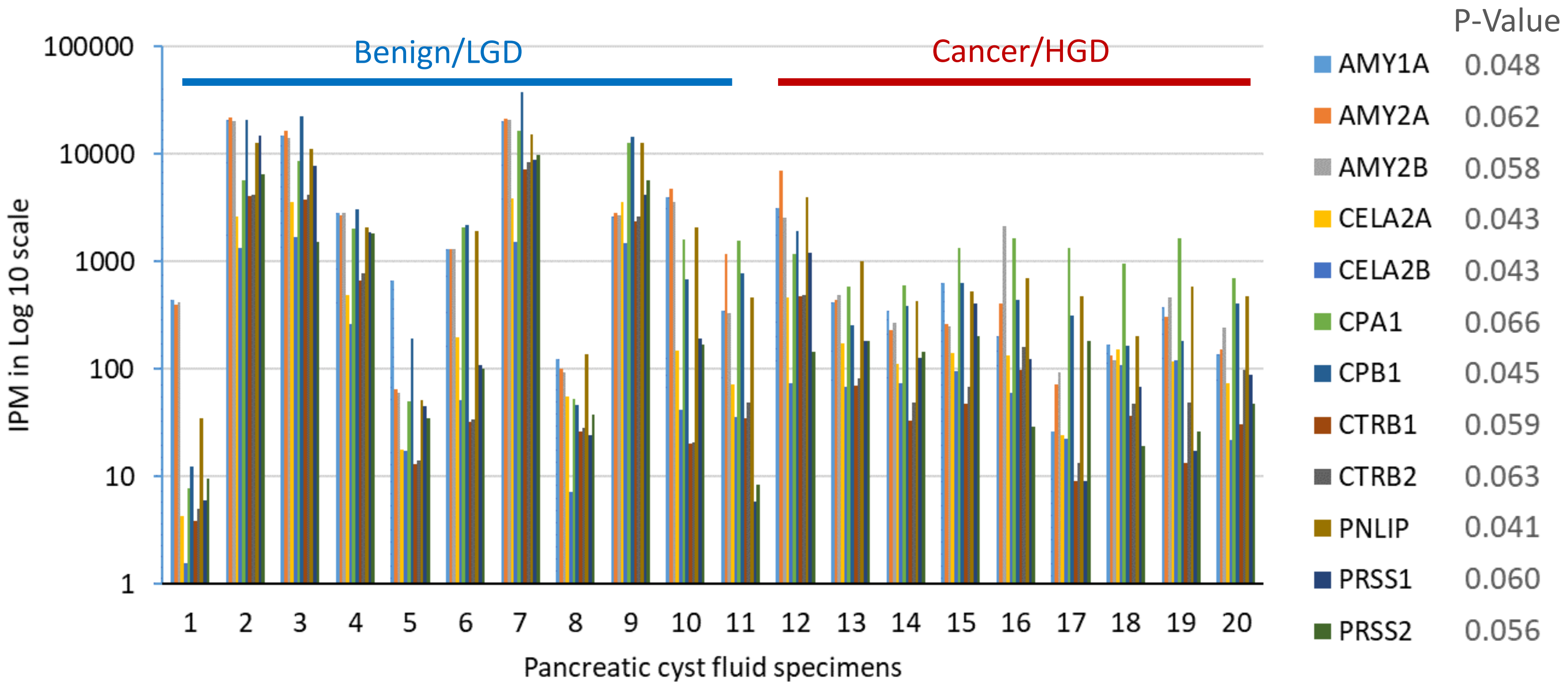

Supplemental Figure 3. The levels of individual enzymes in the pancreatic cyst fluid specimens.

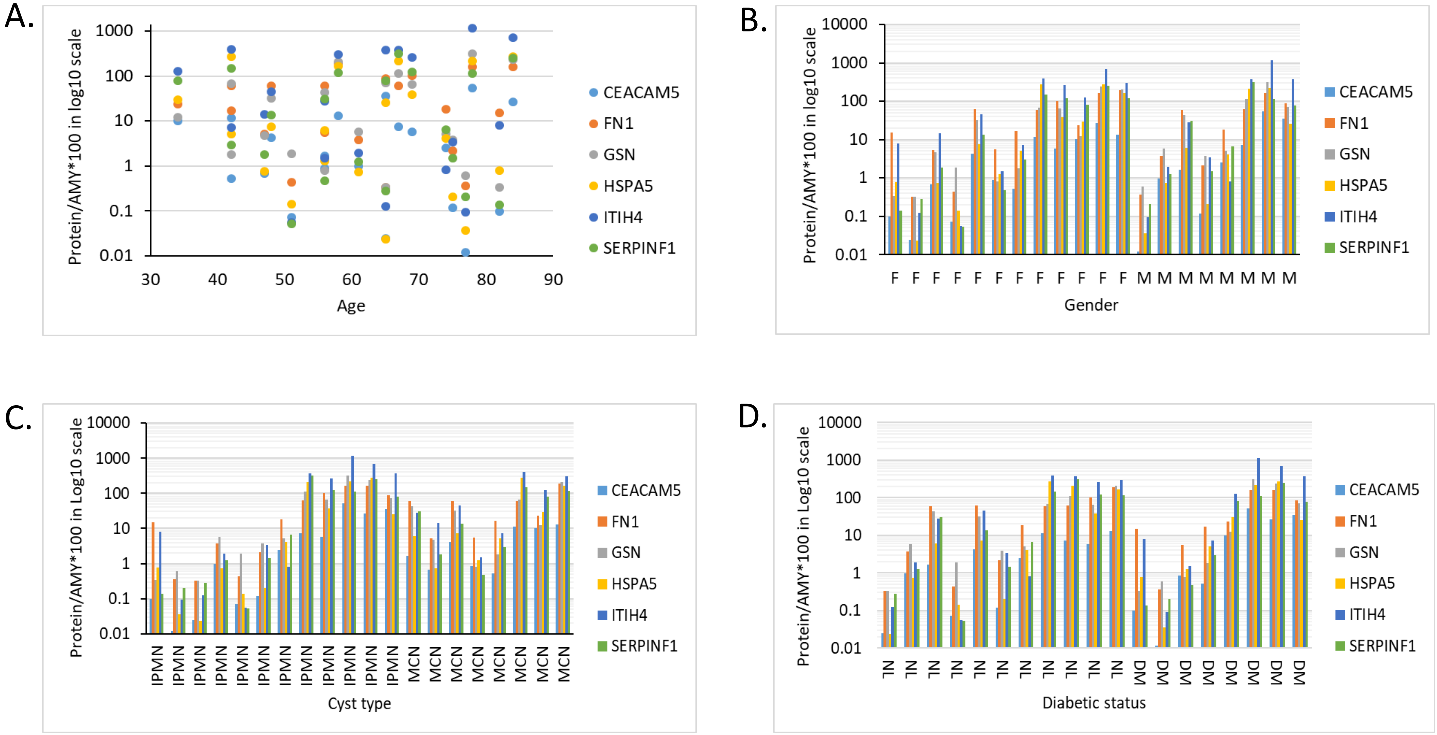

Supplemental Figure 4. The quantification of amylases-normalized CEACAM5, FN1, GSN, HSPA5, ITIH4 and SERPINF1 versus patients' age (A), gender (B), mucinous cyst types (C), or diabetic status (D). No significant correlations were observed between ratio-based quantifications and the patients age, gender mucinous cyst types, or diabetic status.

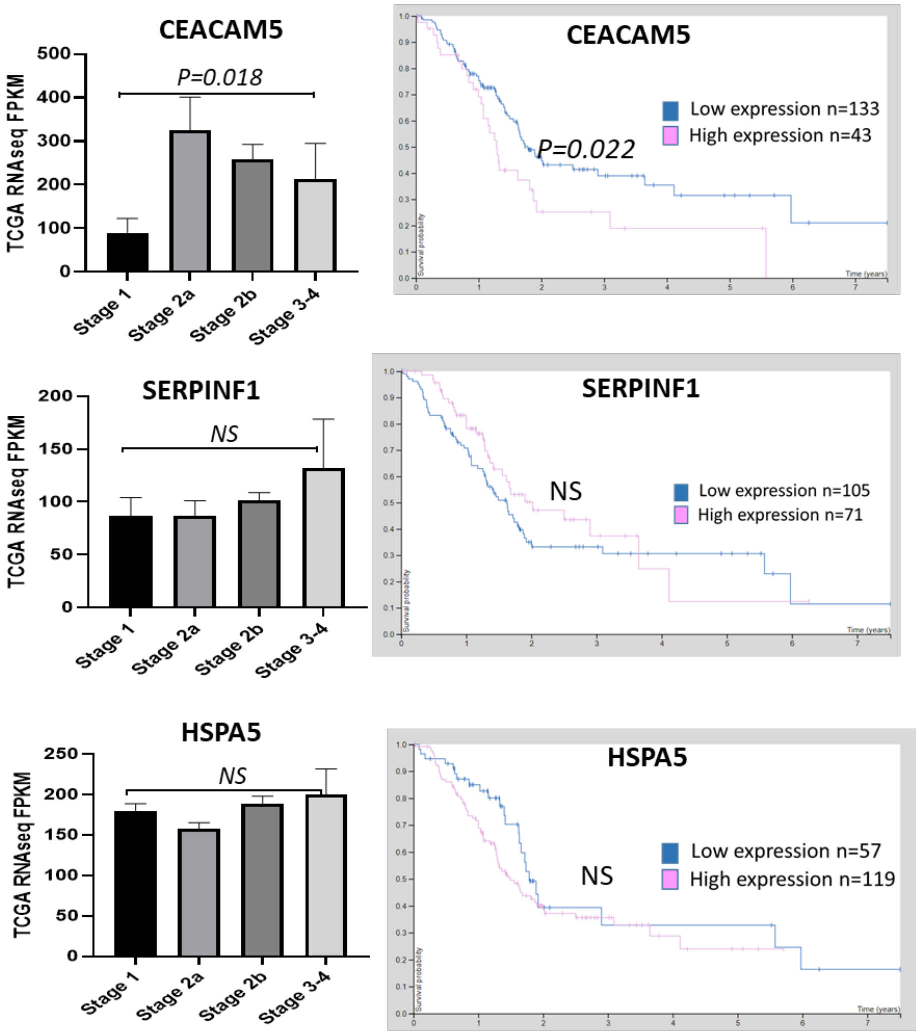

Supplemental Figure 5. Tissue RNA expression analysis in pancreatic cancer using TCGA database for CEACAM5, SERPINF1 and HSPA5. Using the TCGA RNA-seq dataset available from v19.1 ProteinAtlas.org, the RNA expression of CEACAM5, SERPINF1 and HSPA5 were evaluated, and only CEACAM5 was significantly linked to tumor stages and/or patient survival time. NS: statistically non-significant.
