# Supplementary material for: Proteome heterogeneity and malignancy detection in pancreatic cyst fluids
Source: Clin Transl Med. 2021 Aug 23;11(8):e506. doi: 10.1002/ctm2.506 (PMC8382978; doi:10.1002/ctm2.506)
Supplement: Supplementary file 2 — Supporting information [file CTM2-11-e506-s001.pdf]

Supplemental Information

Materials and Methods

Pancreatic cyst fluid specimens. The study was approved by the Institutional Review Boards at the University of Pittsburgh, University of Washington, University of California at Los Angeles, University of Texas Health Science Center at Houston, and Baylor College of Medicine. Informed consent was obtained from each patient for pancreatic cyst fluid collection. The pancreatic cyst fluid specimens were collected with IRB approval and under HIPAA compliant guidelines using standard operating protocols at University of Pittsburgh and UCLA. The case-control cohort from University of Pittsburgh comprised 12 IPMNs and 8 MCNs, including 9 cases with histologically confirmed carcinoma or high-grade dysplasia (HGD) and 11 cases with benign or low-grade dysplasia (LGD) (Supplemental Table 1). The subjects in these study cohorts are highly annotated with detailed demographic characteristics and clinical features. The samples were stored at -80˚C until analysis.

Proteomic analysis. Protein concentration was measured in each pancreatic cyst fluid sample using BCA assay. Fifty microgram of proteins were extracted from each sample, reduced by 10 mM dithiothreitol at 50 ˚C for 1 hr and alkylated by 25 mM iodoacetimide at room temperature in the dark for 30 min. The samples were digested with sequencing grade modified trypsin at 1:30 ratio (w:w) at 37 °C for 18 h. The samples were dried down and re-suspended in 50 µl 0.1% formic acid for MS analysis.

The samples were blinded and analyzed in a random order. The LC MS/MS system includes a Q Exactive™ Plus mass spectrometer (ThermoFisher Scientific) coupled with a nanoACQUITY HPLC (Waters, Milford, MA, USA). The samples were first loaded onto a trapping column (100 µm × 3 cm) then separated with an analytical column (75 µm × 30 cm). The trapping column and the analytical column were packed with ProntoSIL 120 Å-5 µm-C18 AQ beads (Mac-Mod, Chadds Ford, PA, USA). The analytical column was house-made with a tip pulled with a Laser Fiber Puller P-2000 (Sutter Instruments, Novato, CA, USA) at the end of the column. The sample was loaded onto the trapping column with 98% Buffer A (0.1% formic acid in water) and 2% Buffer B (0.1% formic acid in acetonitrile) at a flow rate of 2 µl/min for 10 min, and separated by a linear gradient from 5 to 30% B for 90 min, followed by flushing with 80% B for 10 min and equilibration with 2% B for 20 min. The LC gradient lasted 120 min with a flow rate of 0.3 µl/min. Electrospray ionization was operated in a positive mode at a voltage of 2.1 kV. Data-dependent acquisition (DDA) was performed. The survey scan was done with 70,000 resolution at 200 m/z from 400 to 1200 m/z with an AGC target of 1e6 and max injection time of 100 ms. The precursors were isolated in the quadrupole within an isolation window of 1.6 m/z. The top 20 monoisotopic masses with 2 to 4 plus charges were selected with a minimum intensity threshold of 5e4, then fragmented by higher energy collisional dissociation (HCD). The DDA cycle time was ~3 sec.

Data analysis. The MS data was searched against the UniProt human protein database for peptide/protein identification using the Comet algorithm [1] embedded in the Trans-Proteomic Pipeline [2]. Carbamidomethylation of cysteine was set as fixed modification, and oxidation of methionine was set as variable modifications. The peptide assignment was validated with PeptideProphet [3], and a probability score in correspondence with an FDR of 0.01 was applied to filter the peptides. The Skyline software [4] was used for quantitative analysis of the DDA data. The composite spectral library was built using all of the DDA data collected from the samples analyzed. Quantification was made at MS1 level using the sum of the first 3 monoisotopic peaks. The abundance of each peptide was normalized to total ion current (TIC) and presented as ion per million (IPM) using the following formula: Normalized Intensity (IPM) = Peptide Intensity / TIC * 1000000. Protein quantification was achieved by summation of the normalized intensities of the corresponding peptides.

Statistical analysis. Principal component analysis (PCA) was performed using Graphpad software. PCs were selected based on parallel analysis using Monte Carlo simulations on random data of equal dimension to the input data, and calculating eigenvalues for all the resulting PCs. The software default value of 1000 simulations was selected. A binary logistic regression model was used to conduct the receiver operating characteristic (ROC) analysis with 95% confidence interval using Graphpad software. The performance of markers were evaluated by the ROC curve and the area-under-curve (AUC) value. A p-value ≤ 0.05 was considered as statistical significance. The protein correlation analysis was computed using nonparametric Spearman correlation with 95% confidence interval. Spearman's correlation coefficient R is interpreted as follows: 0.3-0.5 fair, 0.5-0.7 moderate, 0.7-0.9 very strong, 1 perfect.

References

1. Eng JK, Jahan TA, Hoopmann MR. Comet: an open-source MS/MS sequence database search tool. Proteomics 2013;13:22-24.
2. Deutsch EW, Mendoza L, Shteynberg D, et al. Trans-Proteomic Pipeline, a standardized data processing pipeline for large-scale reproducible proteomics informatics. Proteomics Clin. Appl 2015.
3. Keller A, Nesvizhskii AI, Kolker E, et al. Empirical statistical model to estimate the accuracy of peptide identifications made by MS/MS and database search. Analytical Chemistry 2002;74:5383-5392.
4. Maclean B, Tomazela DM, Shulman N, et al. Skyline: an open source document editor for creating and analyzing targeted proteomics experiments. Bioinformatics 2010;26:966-968.
